# Ectopic Calcification as Discernible Manifestation in Neonates with Pseudohypoparathyroidism Type 1a

**DOI:** 10.1155/2009/931057

**Published:** 2009-08-03

**Authors:** Masanori Adachi, Koji Muroya, Yumi Asakura, Yoichi Kondoh, Jun Ishihara, Tomonobu Hasegawa

**Affiliations:** ^1^Department of Endocrinology & Metabolism, Clinical Research Institute, Kanagawa Children's Medical Center, Yokohama 232-8555, Japan; ^2^Department of Pediatrics, Matsuyama Red Cross Hospital, Matsuyama 790-8524, Japan; ^3^Department of Pediatrics, Yokohama Municipal Citizen's Hospital, Yokohama 240-8555, Japan; ^4^Department of Pediatrics, Medical School Keio University, Tokyo 160-8582, Japan

## Abstract

The diagnosis of pseudohypoparathyroidism type 1a (PHP1a) is challenging, because both the osteodystrophy, such as brachydactyly and round face, and the symptomatic hypocalcemia usually develop beyond infancy. Although ectopic calcification may be an early sign of PHP1a, there are no systematic reviews regarding the time of its appearance. We here report on two PHP1a patients who presented with subcutaneous calcification in neonatal period.

## 1. Introduction

Pseudohypoparathyroidism type 1a (PHP1a) is a genetic disorder caused by maternally inherited mutations of GNAS gene, which encodes the *α*-subunit of the stimulatory G protein Gs [1, 2]. Common feature in PHP1a includes Albright hereditary osteodystrophy (AHO) such as brachydactyly [3] and resistance to multiple Gs protein-coupled hormones (e.g., parathyroid hormone (PTH) and thyrotropin) [1]. Individuals with GNAS mutation on paternally inherited alleles manifest only AHO, a variant termed pseudopseudohypoparathyroidism (pseudo-PHP) [4]. The diagnosis of PHP1a often delays due to the high variability of the age at first appearance of characteristic clinical signs [5]. Here we report on two PHP1a patients with GNAS mutations, who were found to have subcutaneous calcification during neonatal period.

## 2. Patient 1

**Figure 1:**
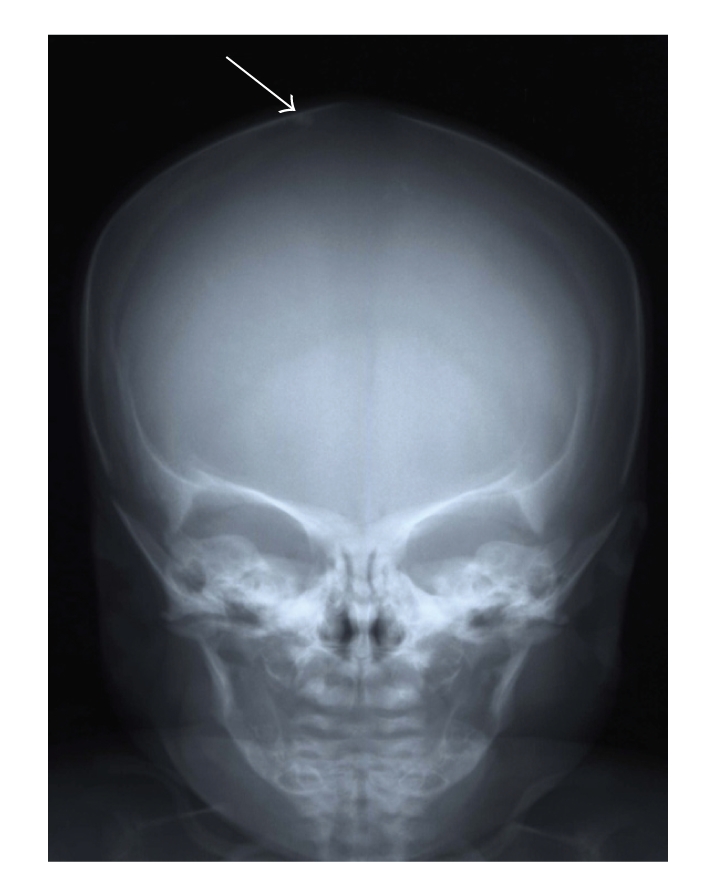
Skull radiograph of patient 1 taken on 13 days of age. White arrow indicates the calcification at right parietal region.

A male baby was born to nonconsanguineous Japanese parents after an uneventful pregnant course. He was delivered by Caesarian section because of bradytocia at his 36 weeks of gestation with his weight measuring 2545 g. Asphyxia was absent. At his 3 days of age, he developed vomiting and hypothermia and was found to have hypocalcemia (5.6 mg/dL). With parenteral infusion of calcium preparation, the above symptoms resolved soon. Calcium supplementation could be withdrawn in a month. By 13 days of age, a subcutaneous nodule at right parietal region was noticed, which was thought to be an ectopic calcification by skull radiograph (Figure 1). Intracranial calcifications, particularly those in basal ganglia, were not found by CT scanning. Despite normocalcemia, his plasma level of intact PTH had been elevated continuously (55–460 pg/mL), which raised the tentative diagnosis of PHP1a. Definite diagnosis, however, was withheld because AHO phenotype was not evident at that time except for ectopic calcification. Because of hypothyroidism, thyroid hormone supplementation was initiated at his 1 year of age. Clinical reevaluation at 4 years of age revealed BMI of 26.8 (above 97th centile, according to the Japanese national survey data [6]), brachydactyly, and round face. His calcium level was still normal (9.0 mg/dL). GNAS gene analysis, under the approval of institutional review board and written informed consent from the parents, revealed the recurrent mutation [7] of 565-568delGACT (189Dfr) in heterozygous state. His mother had short stature of 145 cm (−2.5SD for Japanese women) and brachydactyly, which led to the presumed diagnosis of pseudo-PHP. She, however, did not request DNA analysis.

## 3. Patient 2

**Figure 2:**
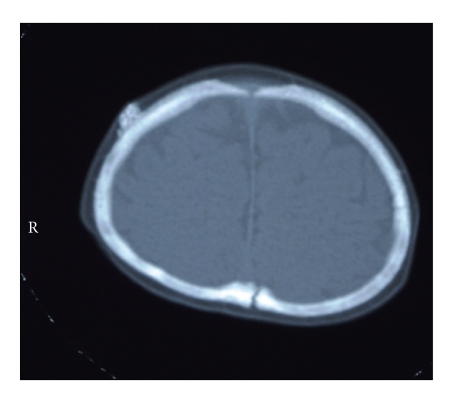
Skull CT image of patient 2 taken at 7 months of age. Calcification at right parietal region is evident.

Patient 2 is a Japanese boy who was born at 36 weeks of gestation by Caesarian section with his weight 2892 g. His elderly brother has ceased at day 1 because of enteric necrosis of unknown origin. His mother had been treated for threatened abortion by magnesium preparation for a month before delivery. Although asphyxia was absent, he developed respiratory distress and hypoglycemia soon after birth, both of which subsided spontaneously. At 2 days of age, hypocalcemia of ionized calcium of 0.81 mmol/L was noticed, which warranted calcium supplementation for about a month. From 9 days of age, he was treated for hypothyroidism. Around 2 weeks of age, a subcutaneous hard nodule was recognized at the right parietal region, CT scanning of which revealed it to be an ectopic calcification (Figure 2). At 6 months of age, brachydactyly and round face were noticed, which raised the suspicion of PHP1a. In addition, several hard nodules less than 10 mm in diameters were present in his back and thigh, which seemed subcutaneous calcifications. Although hypocalcemia had been absent, his PTH level was found to be high (134 pg/mL) after 1.5 year of age. His developmental quotient determined at his 1.5 years of age was 70. GNAS analysis of the family was performed under the approval of institutional review board and written informed consent from the parents. The patient and his mother, who is 140 cm tall (−3.4SD), had heterozygous nonsense mutation (W261X), whereas GNAS of his father, who is 160 cm tall (−1.9SD), was wild type.

## 4. Discussion

During infantile period, the clinical manifestations of PHP1a are usually subtle and, therefore, tend to be easily overlooked. In a case series study, Gelfand et al. [5] reported that brachydactyly in PHP1a was rarely noted in the first 3 years of life, although it was eventually recognized in all 12 patients. In addition, they found that symptomatic hypocalcemia developed later in childhood and that none required calcium and/or calcitriol administration in the first year of life [5].

Subcutaneous calcifications and/or ossifications have also been considered to be one of the constituents of AHO [3]. In a recent study [7], the average of heterotopic ossifications in 12 patients with PHP1a who attended orthopedic clinic was reported to be 10 in number, and no specific tendency was observed regarding the location of ossifications. Gelfand et al. [5] found that ectopic calcifications, next to hypothyroidism, were the second most referral reason in infants with PHP1a. However, detailed evaluation regarding the onset of ectopic calcifications has not been made, and the early onset of ectopic calcifications with AHO has been only anecdotally described [8, 9].

In our patients, subcutaneous calcifications were noticed no later than 2 weeks of age, and they might be already present shortly after birth. As long as we could search in literature, they may be the first cases of molecularly proven PHP1a who developed neonatal-onset subcutaneous calcifications. If this manifestation was paid more attention, it might lead to earlier diagnosis, especially in patient 2. Thus, pediatricians and neonatologists should keep in mind that ectopic calcifications may be the early discernible manifestation of PHP1a.

Subcutaneous calcifications must be distinguished from other conditions. Ectopic calcifications are most frequently reported after subcutaneous fat necrosis following extravasation of calcium gluconate. It can be seen also in hyperparathyroidism, sarcoidosis, and dermatomyositis, and so forth. In addition, paternally inherited GNAS mutation may cause a rare disease termed progressive osseous heteroplasia (POH) [7]. Subcutaneous ossifications in POH are accompanied with ossifications in cutaneous and in deep connective tissues including muscle. Fibrodysplasia ossificans progressive (FOP) is another condition manifesting heterotopic ossification [10]. Patients with FOP usually present with congenital malformations of the great toes, and the average age at onset of ossification was reported to be 5 years [10].

In conclusion, it should be emphasized that ectopic calcifications in neonates may indicate the systemic disorders.
